# THE ROLE OF SOCIABILITY IN OLDER ADULTS' ADAPTATION TO COVID-19

**DOI:** 10.1093/geroni/igac059.895

**Published:** 2022-12-20

**Authors:** Katherine Fiori, Amy Rauer, Christina Marini, Christine So, Amna Khan

**Affiliations:** Adelphi University, Garden City, New York, United States; University of Tennessee Knoxville, Knoxville, Tennessee, United States; Adelphi University, Garden City, New York, United States; Adelphi University, Garden City, New York, United States; Adelphi University, Garden City, New York, United States

## Abstract

Some older adults may be particularly sensitive to the negative effects of social disruptions due to COVID-19 (Tyrrell & Williams, 2020). For example, the unique circumstances of the pandemic may have made greater sociability a liability (Wijngaards et al., 2020). The current study used a community sample of 136 older adults (M age = 67.77, range 50-91; 69.3% females; 93% White) to explore whether sociability moderated links between disruptions to contact with friends and family and indicators of mental health. Using a series of hierarchical linear regressions, we found that sociability moderated the association between disruptions in family interactions and depressive symptoms. For more sociable individuals, disruptions in family interactions were more strongly positively associated with depressive symptoms. Given the potential for future social disruptions due to COVID-19, our findings point to the importance of considering the role of sociability in developing interventions targeting older adults.

